# Digital and immersive approaches to anatomy education: a pilot comparative study of CI, VR, and hybrid learning in implant planning

**DOI:** 10.1186/s12909-026-09010-3

**Published:** 2026-03-19

**Authors:** Sakarat Nalampang, Jirawit Yanchinda, Sangsom Prapayasatok, Arnon Charuakkra, Kreetha Kaewkhong, Jorma Järnstedt

**Affiliations:** 1 Department of Oral Radiology, Faculty of Dentistry, Chiang Mai University, Chiangmai, Thailand; 2 College of Arts, Media and Technology, Chiang Mai University, Chiangmai, Thailand; 3 Department of Curriculum Teaching and Learning, Faculty of Education, Chiang Mai University, Chiangmai, Thailand; 4 Department of Radiology, Tampere University Hospital, Wellbeing Services County of Pirkanmaa, Tampere, Finland; 5 Faculty of Medicine and Health Technology, Tampere University, Tampere, Finland

**Keywords:** Dental education, Virtual reality, Hybrid learning, Computer interface, CBCT, Maxillofacial anatomy, Implant planning, Knowledge retention

## Abstract

**Background:**

A solid understanding of anatomy is fundamental in dental education, and accurate interpretation of maxillofacial structures is essential across clinical contexts. Implantology is an example of a complex procedure that undergraduate students learn theoretically before supervised practice. Cone beam computed tomography (CBCT) provides the necessary radiological information, typically explored through conventional computer interfaces (CI). These non-immersive interfaces support diagnostic interpretation but may offer limited spatial engagement. Virtual reality (VR) provides an immersive 3D environment that may enhance conceptual understanding and long-term retention. Comparative evidence across CI, VR, and hybrid CI + VR modalities in undergraduate dental anatomy and implantology education remains limited.

**Methods:**

Twenty undergraduate dental students were randomly assigned to CI, VR, or CI + VR following a baseline lecture. Learning outcomes were assessed using a validated rubric at Pre-test (knowledge, understanding), Post-test (knowledge, understanding, application), and one-month Follow-Up. Quantitative outcomes were analysed using mean scores, mean differences, percentage change, and retention metrics. Complementary qualitative insights were obtained through short semi-structured interviews focusing on usability, visualization clarity, and perceived educational value.

**Results:**

All modalities improved performance in at least one domain. In Knowledge, mean scores increased from 2.00 to 2.71 in CI (*p* = 0.008), from 1.86 to 2.29 in VR (*p* = 0.078), and from 1.83 to 2.50 in CI + VR (*p* = 0.025). In Understanding, means increased from 4.57 to 6.00 in CI (*p* = 0.008), from 3.71 to 5.71 in VR (*p* = 0.004), and from 5.00 to 6.00 in CI + VR (*p* = 0.076). At Follow-Up, Knowledge means were 2.28 (CI), 2.00 (VR), and 2.33 (CI + VR), and Understanding means were 5.70 (CI), 5.70 (VR), and 5.60 (CI + VR). Application scores increased across all groups (CI: 6.00→6.42; VR: 4.71→5.55; CI + VR: 4.50→4.98). Interviews indicated that CI was valued for diagnostic precision, VR for immersive 3D visualization, and CI + VR for combining accuracy with experiential learning.

**Conclusions:**

Each instructional modality improved learning performance, but with distinct strengths. CI supported diagnostic precision, VR maximized conceptual understanding and retention, and CI + VR offered the most balanced gains with the strongest Knowledge stability. Qualitative findings aligned with these patterns, highlighting CI’s clarity, VR’s immersive engagement, and CI + VR’s combined benefits. Immersive and hybrid technologies therefore represent valuable complementary strategies for enhancing maxillofacial anatomy learning in dental education.

**Supplementary Information:**

The online version contains supplementary material available at 10.1186/s12909-026-09010-3.

## Background

Safe and effective patient care begins with a solid understanding of anatomy [1]. In medical and dental education, students first encounter anatomy through cadaver dissection, models, and lectures, gradually progressing to supervised patient contact [2]. As their studies advance, they encounter topics that are not practiced under supervision; demanding surgical procedures, for example, are excluded from basic training but must still be taught theoretically. Instruction therefore relies heavily on traditional lectures and PowerPoint slides, which provide foundational knowledge but depend on two‑dimensional (2D) representations. These methods may place considerable demands on spatial cognition and therefore hinder deeper conceptual understanding [3]. Integrating radiology and radiological planning into undergraduate curricula offers a feasible way to bridge this gap [4]. By providing three‑dimensional (3D) visualization and clinically relevant planning tools, educators can enhance spatial reasoning and procedural comprehension, equipping students with a stronger theoretical basis that supports later supervised training and the gradual development of operative competence. This need for spatially demanding knowledge is particularly evident in implant dentistry, where safe positioning requires precise anatomical understanding.

Dental implants are artificial roots anchored in the jawbone to support crowns or bridges. Implant placement exemplifies these challenges, as safe positioning requires precise assessment of surrounding anatomical structures. Inadequate spatial understanding may lead to complications such as nerve injury, bleeding, malposition, or mandibular fracture [5–9]. These challenges highlight the need for advanced radiological planning methods.

Anatomical features of the jaws impose significant constraints: in the posterior maxilla, the sinus and nasal cavities restrict implant positioning; in the mandible, the mandibular canal and mental foramen define critical boundaries; and in anterior regions, alveolar ridge morphology and limited bone volume may further complicate placement. Thorough preoperative planning is therefore essential, typically supported by cone beam computed tomography (CBCT), which provides radiological 3D visualization of bone dimensions, dental roots, and adjacent structures (10). Digital workflows increasingly integrate CBCT data with computer interfaces (CI), enabling clinicians to simulate implant placement, measure distances to critical structures, and design surgical guides [10–12]. While computer interfaces (CI) provide diagnostic rigor and accessibility, their reliance on two-dimensional screens constrains spatial immersion and limits the learner’s ability to contextualize anatomical relationships. Virtual reality (VR) refers to computer-generated three-dimensional environments, typically delivered through head-mounted displays and related input. By contrast to CI, VR has been shown to enhance spatial perception and comprehension, and—when appropriately designed—can reduce extraneous cognitive load while supporting deeper conceptualization of anatomy [13–17]. These technological contrasts underscore the distinctive demands of dentistry.

Dentistry represents a distinctive intersection of science, handicraft, and permanence: diagnostic accuracy must be paired with fine motor skill and irreversible interventions within the oral cavity. This combination makes radiological anatomy education particularly challenging and underscores the need for innovative instructional approaches.

In dental education, radiological anatomy has increasingly been taught using digital platforms that extend beyond traditional lectures. Computer interfaces with CBCT software have been employed to familiarize students with three-dimensional datasets and implant planning procedures [18]. Virtual reality applications have also been introduced as immersive tools for teaching dental anatomy, offering interactive environments that enhance spatial reasoning and procedural comprehension [19, 20]. Narrative reviews further highlight the integration of CBCT, VR, and related technologies into dental diagnostics and education [21]. These approaches demonstrate the growing role of radiology-based technologies in dental curricula, yet comparative evidence on their relative effectiveness remains limited. yet comparative evidence on their relative effectiveness remains limited. The present study addresses this gap by directly evaluating CI, VR, and hybrid CI + VR modalities in the context of maxillofacial anatomy and implant planning.

Beyond dentistry, immersive technologies have demonstrated educational benefits across medical fields. In surgery, VR simulation has been shown to improve technical skills such as instrument handling and procedural accuracy, while also strengthening clinical decision-making [13]. In radiology, VR enhances spatial understanding of complex anatomical structures, for example in neurovascular and thoracic imaging, where 3D visualization supports diagnostic precision [14]. Nursing education has adopted VR to facilitate procedural rehearsal and teamwork, improving confidence and coordination in simulated clinical scenarios [15]. Medical anatomy teaching has likewise reported gains in student engagement and retention when immersive visualization is employed, with learners demonstrating better recall of spatial relationships compared to traditional methods [16, 17]. Together, these cross-disciplinary findings highlight the broader relevance of VR and CI as pedagogical tools in medical and dental education.

While immersive technologies demonstrate clear educational benefits, contemporary medical and dental curricula face a growing imbalance: the volume of essential knowledge has expanded considerably, while available study time has remained largely fixed [22–24]. This mismatch places increasing demands on students, who must assimilate complex information under significant time constraints. Innovative instructional approaches—such as immersive visualization, simulation, and digital workflows—offer potential solutions by enhancing efficiency, reducing cognitive load, and supporting deeper conceptual understanding. By optimizing how knowledge is delivered rather than extending study hours, educators can help students meet rising expectations without compromising comprehension or retention. Against this backdrop, we designed the present study to evaluate how different digital modalities can address these curricular challenges.

The aim of this study was to evaluate the effectiveness of computer interfaces (CI), virtual reality (VR), and a hybrid CI + VR model in teaching implant-related dental and complex maxillofacial anatomy to undergraduate dental students. The rationale was to address curricular challenges posed by expanding knowledge demands under fixed study time, by testing whether immersive visualization and digital workflows could enhance knowledge acquisition, conceptual understanding, and clinical application. In addition, student perceptions were explored to provide insight into how these modalities influence both cognitive and experiential aspects of learning. As a preliminary investigation, the findings are intended to inform future multi-institutional studies on the integration of digital and immersive technologies into dental curricula.

## Materials and methods

### Study design

This pilot study employed a pre–post design to evaluate the effectiveness of three instructional modalities—Computer Interface (CI), Virtual Reality (VR), and a hybrid CI + VR approach—on undergraduate dental students’ learning of maxillofacial anatomy and implant planning. Cone-beam computed tomography (CBCT) served as the imaging modality throughout all instructional activities.

### Participants

Twenty third-year dental students from the Faculty of Dentistry, Chiang Mai University, voluntarily participated. Inclusion criteria were enrolment as third-year dental students with completed coursework in anatomy and radiographic interpretation. Exclusion criteria were prior experience with CBCT diagnostic software in CI or VR environments, or reported inability to use the VR headset. As pre-clinical students, they had not yet entered clinical training and had no prior exposure to implant planning or related clinical procedures.

Initially, 21 students were enrolled and allocated equally (*n* = 7 per group) using simple randomization without stratification or block procedures, and allocation concealment was not implemented. During the study, one participant withdrew due to a physical limitation related to the VR headset, leaving 20 participants in the final analysis. Recruitment of a replacement was not possible due to the study protocol, resulting in one group containing six participants.

Baseline grade point averages (GPAs) were collected to ensure academic comparability across groups:

-  CI group: *n* = 7, mean GPA = 3.60
-  VR group: *n* = 7, mean GPA = 3.42
-  CI + VR group: *n* = 6, mean GPA = 3.70

At Chiang Mai University, GPA is reported on a 0–4 scale, with 4.0 representing the maximum possible score. Observed GPAs ranged from 3.20 to 3.90 across participants. The sample size was determined pragmatically based on voluntary recruitment within a single class cohort, reflecting feasibility constraints of this pilot study rather than formal power calculation. While minor GPA differences were observed across groups, these were not considered during randomization and were identified only after allocation. As all participants were of similar age, educational level, and had no prior experience with CI or VR tools, the groups were regarded as comparable for the purposes of this pilot study.

### Patient data

Two CBCT datasets were used: one for anatomical landmark review and implant planning exercise, and another for the clinical simulation task. Both datasets consisted of pseudonymized scans with a field of view of 8 × 8 cm and a voxel size of 0.2 mm. Datasets were selected from routine clinical archives to represent typical implant planning cases. CI, VR and CI + VR groups used the same datasets to ensure comparability.

### Instructional conditions

#### CI Group

Training was conducted using a conventional CI workstation. A Barco MDRC 2224 BL 24-inch DICOM-calibrated monitor (1920 × 1200 pixels) displayed CBCT images across multiple planes (cross-sectional, axial, sagittal, 3D reconstruction, and synthetic panoramic). Image interpretation and virtual implant placement were performed using Blue Sky Plan software (version 4.13, Blue Sky Bio, LLC, USA), which provided measurement tools and controls for rotation, zooming, windowing, and thresholding.

#### VR Group

Students learned through an immersive VR environment using Planmeca Romexis VR (Planmeca Oy, Helsinki, Finland), built on Unity 3D (2021.1). A Meta Quest 3 headset (2064 × 2208 pixels per eye) presented a manipulable 3D anatomical model equipped with dynamic windowing, holographic X-ray rendering, and coronal/sagittal X-ray slicing. Implant models were selected from a predefined library and placed using a dynamic cut-plane tool for internal visualization. The VR application used in this study was the commercially available Romexis VR module, developed by Planmeca Oy. No custom programming was performed beyond standard configuration; the environment was implemented using the vendor’s existing Unity-based framework.

#### CI + VR Group

Students first completed the CI session followed immediately by the VR session, using the same instructional framework.

#### Learning procedure

All participants attended a 40-minute baseline lecture addressing maxillofacial anatomy and key principles of implant placement. In this lecture, students were guided to identify nine key anatomical landmarks in the CBCT region of interest:

1. Mandibular canal
2. Mental foramen
3. Submandibular fossa
4. Genial tubercle
5. Incisive canal
6. Maxillary sinus
7. Nasal floor
8. Apexes of the second premolars
9. Lateral incisors

In addition, students evaluated alveolar crest height and width, as well as bone density. These landmarks and parameters were consistently emphasized across both CI and VR practice sessions to ensure reproducibility of the learning tasks.

After the baseline lecture, students were randomly assigned to one of the following groups:

-  CI group: Guided introduction to the conventional interface (~ 10 min) followed by ~ 10 min of individual practice placing a virtual implant in region #21 (upper left central incisor). Total instructional time: ~20 min.
-  VR group: Guided introduction to the VR environment (~ 10 min) followed by ~ 10 min of individual implant placement practice in region #21. Total instructional time: ~20 min.
-  CI + VR group: Completed CI introduction and practice (~ 20 min) followed immediately by VR introduction and practice (~ 20 min). Total instructional time: ~40 min.

#### Clinical simulation task 

**Fig. 1:**
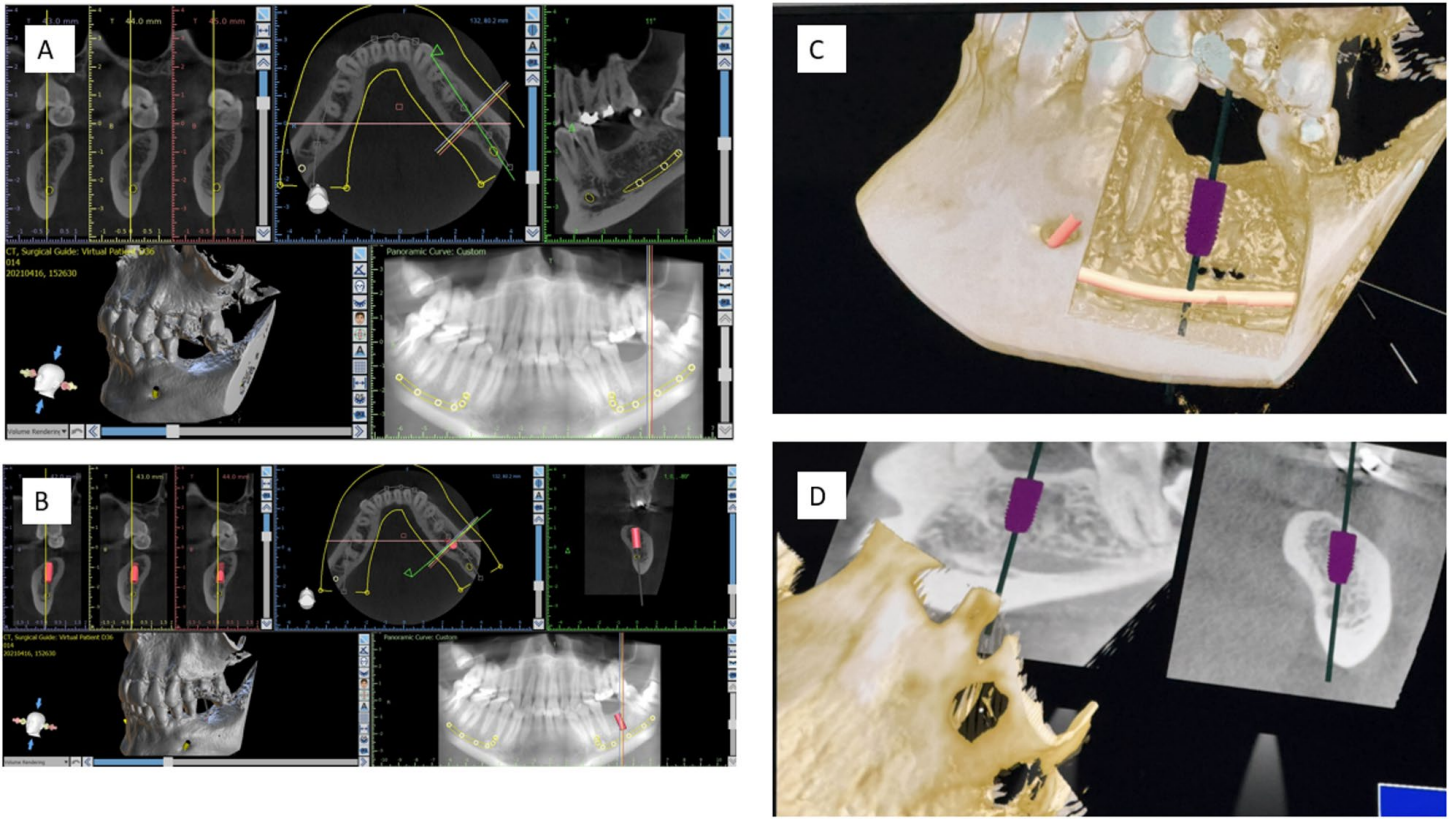
Multimodal views of implant planning in site #36 using CI and VR. Panels show CI and VR. Yellow lines mark the mandibular canal; red in CI and purple in VR indicate virtual implants. **A** CBCT slices in multiple planes with measurement overlays, including cross-sectional, axial, sagittal, 3D, and synthetic panoramic views. **B** Sagittal CBCT view oriented to the implant site. **C** VR-rendered 3D jaw model with implant placement, highlighting spatial orientation and bone contours. **D** VR sagittal and coronal views illustrating implant alignment and anatomical integration

All participants completed a standardized implant planning exercise in the mandibular left first molar region (tooth #36, lower left first molar). Students identified anatomical landmarks, selected implant dimensions, positioned the implant, adjusted angulation, and verified safety margins using the same CBCT dataset in both conditions. In both CI and VR, participants could inspect conventional CBCT slices as well as 3D models of the anatomy, displayed either through a conventional interface (CI) or within a VR environment (VR) (Fig. 1). The time required for the clinical simulation task varied between individuals (typically 5–10 min in both CI and VR), as participants were allowed to proceed at their own pace. Individual time consumption was not measured, as the study focused on learning outcomes rather than task duration.

#### Assessment instruments

##### Quantitative instruments

1. Knowledge and Understanding Test

Structured exam with multiple‑choice and short‑answer items.

2. Clinical Application Test

Two case‑based CBCT interpretation tasks (#37 and #23). 

The full item sets are provided in Supplementary Additional Files 1 and 2 for transparency and reproducibility.

#### Quantitative assessment timeline

Learning outcomes were assessed at three time points using these instruments:

1. Pre test (immediately after the baseline lecture): Knowledge and Understanding were measured with the structured exam to establish baseline performance.
2. Post test (immediately after the clinical simulation): Both test instruments were administered — the structured exam (Knowledge and Understanding) and the CBCT case based tasks (Clinical Application). Clinical Application was introduced here, as participants had no prior procedural experience in CBCT or VR environments.
3. Follow Up (one month later): Both test instruments in identical format to the Post test, enabling retention analysis across all domains.

#### Qualitative assessment instrument

##### Student satisfaction

All 20 participants took part in semi-structured interviews conducted individually two days after the post-test. Each interview lasted approximately 10–15 min and followed a five-question guide addressing perceptions of the learning activity, differences from prior study experiences, preferred aspects, areas needing improvement, and perceived impact on anatomical understanding. For the CI + VR group, two additional prompts were included to explore perceptions of the combined modality. Interviews were audio-recorded, transcribed verbatim, anonymised, and analysed thematically using an inductive, descriptive approach. The full interview guide is provided in Supplementary Additional File 3.

**Fig. 2:**
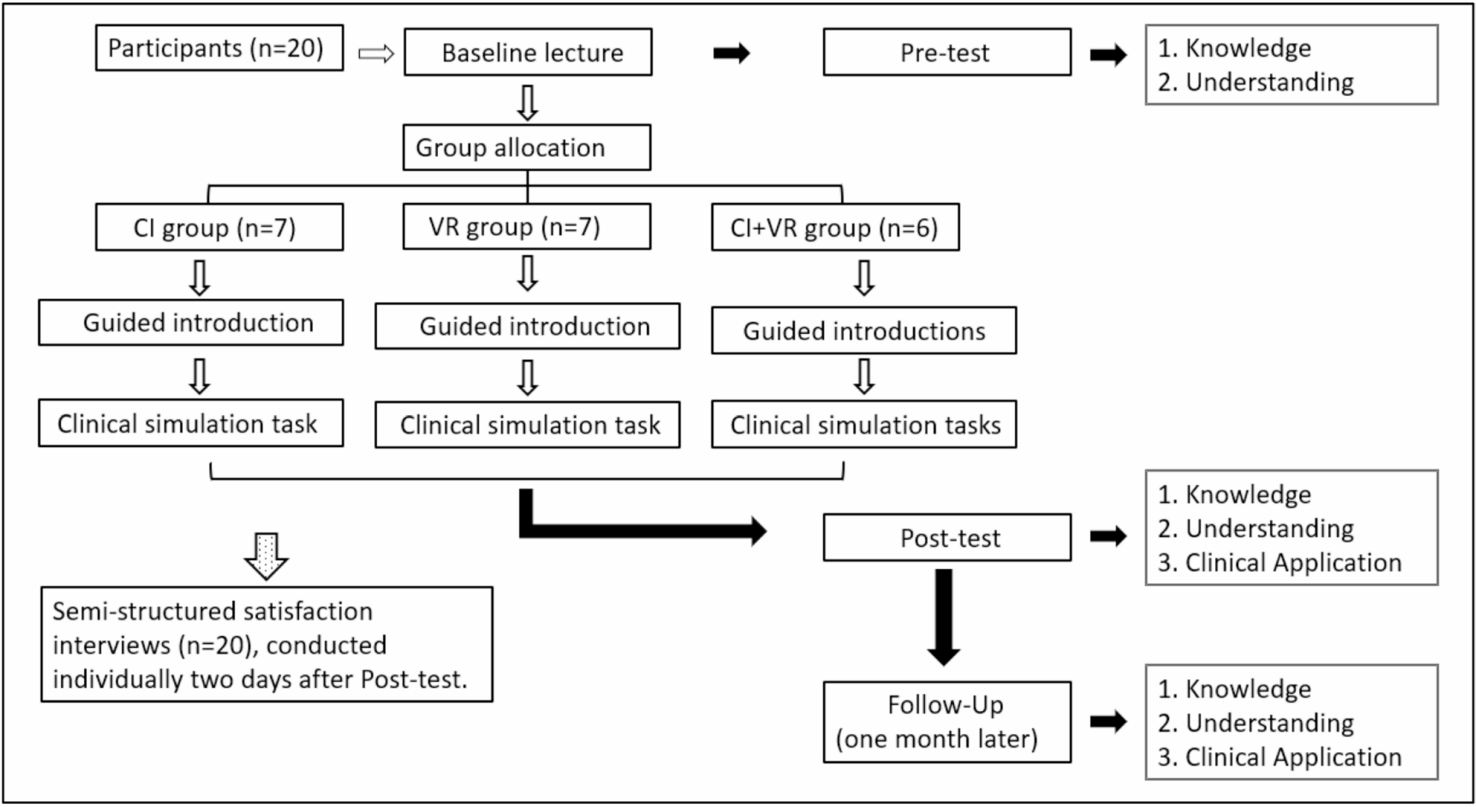
Study timeline

The overall study workflow is summarized in Fig. 2.

Study design flowchart. All participants completed Pre-test (Knowledge, Understanding), Post-test (Knowledge, Understanding, Clinical Application), and Follow-Up (one month later). Semi-structured satisfaction interviews were conducted individually with all 20 participants two days after the Post-test.

### Data analysis

Conversion to a 0–3 rubric was chosen to standardize scoring across domains and facilitate comparison. Weighting factors reflected cognitive complexity, with Clinical Application assigned the highest weight (value = 3). Maximum possible weighted scores were: Knowledge = 3, Understanding = 6, Clinical Application = 9.

Short answer responses were evaluated for accuracy and completeness according to a structured rubric aligned with Bloom’s Taxonomy. The rubric assigned progressive weights to cognitive levels (Knowledge = 1 through Create = 6), and each answer was graded using a three-tier scale: 1 (Incomplete), 2 (Correct but not fully complete), and 3 (Correct and comprehensive). All scoring was conducted by the main researcher, with blind grading to ensure anonymity. Inter rater reliability was not assessed due to the single coder design. This approach ensured consistency across all assessments, though reliance on a single rater may limit objectivity.

Paired samples t tests (two tailed; *p* < 0.05, *p* < 0.01) were applied within each group. Non-significant results were reported as ‘ns.’ No inferential statistical testing was conducted for any Follow Up outcomes; all Follow Up results are descriptive.”

Effect sizes were calculated for all primary within-group comparisons (Pre-test → Post-test, Post-test → Follow-up) using Hedges’ g for paired samples.

Operational definitions: were as follows:

-  % change = (Post-test – Pre-test)/Pre-test × 100
-  % retention = (Follow-Up/Post-test × 100)
-  % memory loss = (Post-test – Follow-Up)/Post-test × 100

While repeated use of identical items may introduce a test–retest effect, this design ensured consistency across phases.

#### Student satisfaction

Transcripts were coded by the main researcher, with codes reviewed by a second researcher to ensure consistency. Themes were identified through iterative discussion and consensus. Formal inter-coder agreement statistics were not calculated given the pilot nature of the study, but coding consistency was ensured through collaborative review.

## Results

### Participants and assessment framework

Twenty undergraduate dental students participated and were allocated to three instructional modalities: conventional instruction (CI; *n* = 7), virtual reality–based learning (VR; *n* = 7), and a hybrid CI + VR approach (*n* = 6). Learning outcomes were evaluated across Knowledge, Understanding, and Clinical Application domains using rubric-based scoring. Knowledge and Understanding were assessed at Pre-test, Post-test, and one-month Follow-Up, while Clinical Application was assessed at Post-test and Follow-Up only. Given the exploratory nature of the study and the limited sample size, analyses were conducted at the group level, with emphasis on observed patterns alongside statistical significance.

A consolidated overview of mean scores across all domains is presented in Supplementary Table S1, while raw individual scores are reported in Supplementary Table S2. Together, these tables enable inspection of variability across participants and instructional groups, ensuring methodological transparency.

### Quantitative analysis

Immediate Learning Gains (Pre‑test → Post‑test).

Results are shown in Table 1.

Across instructional modalities, mean scores increased from Pre-test to Post-test in both Knowledge and Understanding domains. Statistically significant Knowledge gains were observed in the CI group (2.00 → 2.71; +35.50%; *p* = 0.008; Hedges’ g = 0.95, large) and the CI + VR group (1.83 → 2.50; +36.61%; *p* = 0.025; Hedges’ g = 1.12, large). The VR group demonstrated a smaller increase in Knowledge (1.86 → 2.29; +23.12%), which did not reach statistical significance (*p* = 0.078); however, the effect size was moderate–large (Hedges’ g = 0.71).

For Understanding, significant improvements were observed in the CI group (4.57 → 6.00; +31.29%; *p* = 0.008; Hedges’ g = 0.91, large) and the VR group (3.71 → 5.71; +53.91%; *p* = 0.004; Hedges’ g = 1.54, very large). The CI + VR group also showed an increase in Understanding (5.00 → 6.00; +20.00%), although this change was not statistically significant (*p* = 0.076); the effect size was moderate–large (Hedges’ g = 0.79).

**Table 1:** Immediate learning outcomes across instructional modalities. Pre-test and Post-test mean scores (± SD), absolute mean differences, percentage change, p-values, and effect sizes (Hedges’ g) for Knowledge and Understanding across the three instructional modalities (CI, VR, and CI + VR). Statistical analyses reflect within-group comparisons of immediate learning gains. Results are presented to illustrate patterns of improvement and statistical significance within the context of an exploratory study with a limited sample size

Domain	Learning Group	Pre-test Mean ± SD	Post-test Mean ± SD	Mean Difference	% Change	*p*-value	Hedges’ g (Magnitude)
Knowledge	CI	2.00 ± 0.00	2.71 ± 0.49	+ 0.71	+ 35.50%	0.008 (**)	0.95 (Large)
	VR	1.86 ± 0.38	2.29 ± 0.49	+ 0.43	+ 23.12%	0.78 (ns)	0.71 (Moderate–Large)
	CI + VR	1.83 ± 0.41	2.50 ± 0.55	+ 0.67	+ 36.61%	0.025 (*)	1.12 (Large)
Understanding	CI	4.57 ± 0.98	6.00 ± 0.00	+ 1.43	+ 31.29%	0.008 (**)	0.91 (Large)
	VR	3.71 ± 0.76	5.71 ± 0.76	+ 2.00	+ 53.91%	0.004 (**)	1.54 (Very Large)
	CI + VR	5.00 ± 1.10	6.00 ± 0.82	+ 1.00	+ 20.00%	0.076 (ns)	0.79 (Moderate–Large)

Retention and Follow-Up Outcomes (Post-test → Follow-up).

Results are shown in Table 2.

At one-month follow-up, Knowledge scores declined modestly across groups. CI decreased from 2.71 to 2.28 (84.13% retention; Hedges’ g = − 0.48, small–moderate decline), VR from 2.28 to 2.00 (87.72%; g = − 0.34, small decline), and CI + VR from 2.50 to 2.33 (93.20%; g = − 0.35, small decline), with CI + VR showing the smallest decline.

Understanding was largely maintained (Table 2). CI decreased slightly (6.00 → 5.70; 95.00% retention; g = − 0.34, small decline), VR showed no change (5.70 → 5.70; 100%; g = 0.00, no change), and CI + VR showed a small decline (6.00 → 5.60; 93.33%; g = − 0.35, small decline).

Clinical Application scores increased from Post-test to Follow-up in all groups, indicating continued consolidation (Table 2). CI improved from 6.00 to 6.42 (g = 0.09, negligible), VR from 4.71 to 5.55 (g = 0.27, small), and CI + VR from 4.50 to 4.98 (g = 0.11, negligible).

**Table 2:** Learning retention and follow-up performance. Post-immediate and one-month Follow-Up mean scores, retention percentages, and memory-loss values for Knowledge, Understanding, and Clinical Application across instructional modalities. Retention percentages indicate the durability of learning over time, while negative memory-loss values in the Clinical Application domain reflect continued improvement beyond the immediate instructional period. All Follow-Up results are descriptive and were not subjected to inferential statistical testing

Domain	Learning Group	Post-immediate (mean)	1-month (mean)	Retention (%)	Memory Loss (%)	Hedges’ g (Magnitude)
Knowledge Acquisition	CI	2.71	2.28	84.13%	15.87%	–0.48 (Small–Moderate decline)
	VR	2.28	2.00	87.72%	12.28%	–0.34 (Small decline)
	CI + VR	2.50	2.33	93.20%	6.80%	–0.35 (Small decline)
Understanding	CI	6.00	5.70	95.00%	5.00%	0.34 (Small decline)
	VR	5.70	5.70	100.00%	0.00%	0.00 (No change)
	CI + VR	6.00	5.60	93.33%	6.67%	–0.35 (Small decline)
Clinical Application	CI	6.00	6.42	107.00%	–7.00% (increase)	0.09 (Negligible)
	VR	4.71	5.55	117.82%	–17.82% (increase)	0.27 (Small)
	CI + VR	4.50	4.98	110.67%	–10.67% (increase)	0.11 (Negligible)

#### Qualitative analysis

##### Student satisfaction

A total of 20 students were interviewed: 7 from the CI group, 7 from the VR group, and 6 from the CI + VR group. Student interviews provided complementary qualitative insights, highlighting differences in usability, visualization, educational value, and limitations across the three modalities (Table 3).

-  CI: Students described the interface as demanding — “It took me a long time to figure out the interface, and I needed the instructor to guide me” — but valued its diagnostic precision — “The measurement tool is crucial for evaluating whether the depth is appropriate.”
-  VR: Praised for immersive realism — “It felt like handling a real patient” — though some found manipulation difficult — “The object floated around, and for newcomers without guidance it was hard.” Students emphasized its educational value — “Learning by doing gave me confidence” — while noting limitations such as the absence of measurement tools.
-  CI + VR: Students described the hybrid approach as combining clarity with realism — “Cone Beam lets me plan each aspect clearly, while VR makes it feel like actual practice.” Others noted challenges in coordination — “It was tiring to manage both tools smoothly, but together they gave me the most complete picture.”.

These illustrative quotes highlight how the reported themes were grounded in students’ own perspectives.

**Table 3:** Summary of thematic insights from student interviews on CI, VR, and CI + VR modalities

Theme	CI	VR	CI + VR
Usability	Steep learning curve; non-intuitive interface; required instructor guidance	Engaging and intuitive; fun	Combining both tools required extra coordination
Visualisation & Clarity	Precise, structured multi-plane views; strong for measurement accuracy	Immersive 3D visualization; intuitive spatial awareness; “like handling a real patient”	Combines diagnostic precision with immersive contextualization
Educational Value & Engagement	Supported diagnostic knowledge; highlighted clinical relevance of CBCT	Enhanced conceptual understanding and retention; repeatable, accessible alternative to cadaver work; boosted confidence through “learning by doing”	Viewed as optimal blend: CI for accuracy, VR for experiential learning; balanced confidence
Limitations	Case-specific variability; overwhelming interface	headset discomfort; fatigue; lack of measurement tools, system instability, no dual interface	After CI, some students felt tired or less confident entering VR; harder to connect both experiences smoothly

To contextualize the qualitative findings, we also examined the proportion of participants who mentioned each theme. For example, 5 of 7 CI students (71%) described a steep learning curve, 6 of 7 VR students (86%) emphasized immersive realism, and 4 of 6 CI + VR students (67%) noted challenges in coordinating both tools. These proportions illustrate the relative salience of themes across modalities and complement the thematic summary presented in Table 3.

## Discussion

The findings of this study, although situated within dental implantology, align with broader developments in medical and dental education, where immersive technologies integrate analytical precision with experiential learning. Consistent improvements and retention were observed across domains, with negative memory-loss values in Clinical Application reflecting continued consolidation beyond the immediate instructional period. These results highlight distinct strengths of the three learning modalities—Computer Interface (CI), Virtual Reality (VR), and the hybrid CI + VR approach—in supporting knowledge acquisition, conceptual understanding, and durable learning. Taken together, the findings support growing evidence that digital and immersive tools can meaningfully enhance anatomical education and clinical reasoning [16, 25].

CI supported diagnostic knowledge effectively, consistent with prior work showing that structured, image-based instruction reinforces factual learning and radiographic accuracy [26]. Reduced retention and lower Clinical Application performance align with cognitive load theory, which suggests that static or analytically demanding interfaces impose extraneous load that limits schema formation and long-term consolidation [27, 28]. Student feedback citing navigation difficulties and mental integration challenges supports this interpretation. The modest improvement in application over time may reflect gradual maturation of reasoning rather than instructional affordances.

VR demonstrated clear benefits for conceptual understanding and Clinical Application. Students described the immersive experience as “like handling a real patient,” consistent with embodied cognition frameworks that propose interaction with manipulable 3D structures enhances mental model formation [29, 30]. Stable performance at one-month follow-up suggests that VR creates distinctive episodic learning experiences that support retention, in line with findings from immersive anatomy education [16, 31]. Although concerns about headset comfort and technical stability remain, such limitations are typical of early-stage VR platforms and do not diminish the conceptual advantages observed.

A paradox emerged in the CI + VR cohort: students achieved the highest gains in knowledge acquisition, yet their improvement in understanding was more modest than in the CI or VR groups. This pattern is theoretically coherent. Alternating between analytic CI manipulation and immersive VR visualization likely induced a split-attention effect, increasing extraneous cognitive load and hindering schema integration [32]. Qualitative feedback supports this interpretation, with students describing the experience as “tiring to coordinate both tools smoothly” or noting confusion when switching perspectives. According to the expertise reversal effect, instructional support benefits novices only when cognitive demands across modalities are aligned; when interfaces require incompatible processing, performance may stagnate or decline [33]. Thus, the hybrid paradox does not undermine multimodal learning but underscores the need for careful orchestration and interface coherence.

Beyond this paradox, VR’s strong performance underscores its capacity to accelerate comprehension of complex maxillofacial anatomy. Students without prior exposure advanced rapidly to tasks typically reserved for more experienced cohorts. Neurocognitive research provides a plausible explanation: immersive 3D environments activate both dorsal (“where/how”) and ventral (“what”) visual pathways, enhancing spatial integration and structural identification [34]. Colour-rich, manipulable VR environments further strengthen hippocampal encoding and embodied simulation, supporting long-term retention [35]. The perfect retention observed in the VR group for understanding aligns closely with these mechanisms.

The hybrid CI + VR modality combined CI’s precision with VR’s spatial contextualization. Prior research shows that integrating analytic and experiential information strengthens encoding and transfer [36, 37], reflected here in the hybrid group’s superior long-term retention of knowledge. Students valued CI’s measurement accuracy and VR’s ability to clarify spatial relationships. Remaining challenges—such as mismatches between instructor and student VR views—underscore the need for more refined pedagogical and technical integration in future iterations.

Beyond statistical significance, effect size analysis provided additional insight into the magnitude and durability of learning outcomes. Large effect sizes for both Knowledge and Understanding from Pre-test to Post-test across all modalities indicated substantial immediate gains. The VR group showed the largest effect size for Understanding, supporting the view that immersive environments particularly enhance conceptual and spatial learning rather than factual knowledge alone. At one-month follow-up, Knowledge scores declined slightly across groups, consistent with established forgetting curves, while Understanding scores remained stable. Notably, the VR group showed no measurable decline, suggesting stronger long-term retention of conceptual understanding. This pattern should be interpreted cautiously, as ceiling effects in the Understanding rubric may have limited sensitivity to subtle changes, especially in groups with high Post-test scores. For Clinical Application, effect sizes from Post-test to Follow-up were small to negligible, indicating stable procedural performance. Given that participants were pre-clinical students with no prior exposure and no additional practice during follow-up, these findings likely reflect procedural stabilization rather than delayed skill acquisition. Overall, the results highlight differential learning trajectories across domains and underscore the importance of aligning assessment sensitivity with intended learning outcomes in future studies.

Baseline differences also shaped learning patterns. The CI + VR group displayed the highest initial Understanding scores, constraining proportional improvement due to ceiling effects. Similarly, CI students began with high baseline scores, limiting observable gains. In contrast, the VR group—starting lower—showed larger relative gains, consistent with evidence that immersive technologies disproportionately benefit learners with limited prior knowledge [38, 39]. This aligns with the expertise reversal framework and supports VR as an equitable tool for spatially demanding content. Effect size analysis further highlights the strong impact of VR-based learning on conceptual understanding, reinforcing that these gains were meaningful despite baseline differences.”

Long-term retention patterns provide compelling evidence. The VR group uniquely maintained full retention of conceptual understanding at one month, countering the decay predicted by forgetting curve models. The hybrid CI + VR group showed the strongest retention of factual knowledge, suggesting that multimodal reinforcement strengthens declarative memory. All groups improved in application scores at follow-up, which may reflect delayed consolidation, greater familiarity with clinical decision making, or contextual transfer of skills learned during instruction. This improvement is unlikely to reflect additional teaching, as participants had no scheduled implantology instruction or CBCT exposure during the interval. Familiarity with the Post-test format may also have contributed, alongside delayed consolidation.

Student feedback revealed distinct usability challenges. CI was seen as precise but required instructor guidance due to a steep learning curve and non‑intuitive interface, reflecting the broader complexity of CBCT planning software. VR was described as engaging and intuitive, though headset discomfort and missing measurement tools were noted. These differences likely influenced Application scores, as CI supported structured measurement while VR emphasized spatial awareness without quantitative tools. Collectively, these perceptions suggest that usability challenges and feature gaps are not unique to VR but reflect the broader demands of complex digital diagnostic interfaces. In our study design, participants practiced once and completed the test immediately thereafter, so the learning curve could not be systematically evaluated. Future work with repeated sessions could clarify whether adaptation differs between VR and CI.

Our findings align with previous work showing that VR enhances spatial comprehension in CBCT interpretation [18]. While that focus was diagnostic visualization, our study extends to implant-related anatomy. Although the earlier study included a larger sample, our hybrid CI + VR model and emphasis on curricular integration provide complementary insights. VR has also been found effective for teaching root canal anatomy, underscoring its potential for complex structures (19). A systematic review concluded that VR supports dental anatomy education and retention [20], while other work emphasized the broader integration of CBCT, AI, AR, and VR in education and diagnostics [21]. Collectively, these studies reinforce the growing evidence base for VR in dental education, while our work contributes uniquely by introducing a hybrid CI + VR model and explicitly analysing GPA and ceiling effects to clarify proportional learning gains.

These results highlight the broader curricular imbalance between expanding knowledge demands and fixed study time [22–24]. Efficiency gains with VR and hybrid approaches—especially in conceptual understanding and retention—suggest that immersive visualization may help mitigate this mismatch by supporting deeper learning within limited hours. Immersive technologies thus emerge not as novelty tools but as pedagogical environments reshaping engagement with complex anatomy. VR bridges theoretical knowledge and clinical application, CI provides analytical rigor for factual understanding, and hybrid modalities combine benefits through multimodal encoding. Similar patterns are reported in other health professions, where VR enhances surgical skills, radiological comprehension, teamwork, and clinical decision making [16, 30].

As a pilot study, this work offers insight into combining analytical and experiential modalities in anatomy and implantology education. While performance was shaped by software design constraints, the broader implications—particularly VR’s strengths in conceptual learning and hybrid approaches in consolidation—are transferable. Larger multi-institutional studies, diverse datasets, and improved VR infrastructure will be essential for progress. These findings underscore the potential of immersive technologies in dental education, especially in domains requiring spatial reasoning, clinical precision, and durable memory.

### Limitations

This study has several limitations. The findings reflect the instructional affordances of the specific CI and VR software platforms employed, and may not be directly transferable to systems with different interface designs or functionalities. The small sample size and single-institution design restricted statistical power and generalizability. Use of only two CBCT datasets limited anatomical diversity. The VR setup lacked dual headsets, hindering real-time instructor–student alignment. Together, these factors underscore the preliminary nature of the work and highlight the need for larger, multi-institutional studies with varied datasets and improved technical integration.

Furthermore, the absence of a Pre‑test for Clinical Application limits the ability to quantify individual learning gains in this domain. While Post‑test and Follow‑Up scores reflect newly acquired skills and their short‑term retention, the lack of baseline data should be considered a limitation when interpreting these results. In addition, the CI + VR group received approximately double the instructional exposure compared to CI or VR alone, which may represent a potential confound when interpreting group differences.

## Conclusions

This pilot study evaluated the effectiveness of computer interfaces (CI), virtual reality (VR), and a hybrid CI + VR model in teaching implant-related dental and complex maxillofacial anatomy to undergraduate dental students. CI supported diagnostic knowledge, VR enhanced conceptual understanding and stabilized retention, and the hybrid model buffered against knowledge erosion. Student perceptions highlighted improved engagement and spatial comprehension, underscoring the educational value of immersive workflows. These findings suggest that digital and hybrid modalities can help address curricular challenges posed by expanding knowledge demands under fixed study time. In addition, proportional gains were shaped by ceiling effects in groups with high baseline scores, emphasizing the importance of considering initial knowledge levels when interpreting outcomes. Together, these insights provide a rationale for larger multi-institutional studies on the integration of digital and immersive technologies into dental curricula.

### Take-home message

Immersive technologies enrich anatomical education in dentistry. Using implantology as a clinical exemplar, this study shows that CI and VR can be integrated more broadly into medical curricula to strengthen understanding, stabilize retention, and promote equitable learning outcomes.

## Supplementary Information

Supplementary Material 1.

## Data Availability

The datasets used for the image quality analysis were provided by the Wellbeing Services County of Pirkanmaa and Tampere University Hospital. Due to legal and data protection restrictions under Finnish law and the EU GDPR, these datasets are not publicly available.

## Abbreviations

• CBCT: Cone beam computed tomography
• CI: Computer interfaces
• VR: Virtual reality
• GPA: Baseline grade point average
• 2D: Two–dimensional
• 3D: Three–dimensional
