# Supplementary material for: Digital and immersive approaches to anatomy education: a pilot comparative study of CI, VR, and hybrid learning in implant planning
Source: BMC Med Educ. 2026 Mar 19;26:681. doi: 10.1186/s12909-026-09010-3 (PMC13122969; doi:10.1186/s12909-026-09010-3)
Supplement: Supplementary file 1 — Supplementary Material 1. [file 12909_2026_9010_MOESM1_ESM.docx]

Digital and Immersive Approaches to Anatomy Education: A Pilot Comparative Study of CI, VR, and Hybrid Learning in Implant Planning

Sakarat Nalampang^1^, Jirawit Yanchinda^2^, Sangsom Prapayasatok^1^, Arnon Charuakkra^1^, Kreetha Kaewkhong^3^, Jorma Järnstedt^4,5^ \^*^

1. Department of Oral Radiology, Faculty of Dentistry, Chiang Mai University, Chiangmai, Thailand
2. College of Arts, Media and Technology, Chiang Mai University, Chiangmai, Thailand
3. Department of Curriculum Teaching and Learning, Faculty of Education, Chiang Mai University, Chiangmai, Thailand
4. Department of Radiology, Tampere University Hospital, Wellbeing Services County of Pirkanmaa, Tampere, Finland
5. Faculty of Medicine and Health Technology, Tampere University, Tampere, Finland

\^*^Corresponding author:

Jorma Järnstedt

Email: jorma.jarnstedt@pirha.fi

SUPPLEMENTARY

*Supplementary Table S1.*

Group-level mean ± SD values at Pre-test, Post-test, and Follow-Up (1‑month) across Knowledge, Understanding, and Application domains. Absolute changes (Δ Post→Follow-Up) and percentage changes are reported to illustrate trajectories within each instructional modality. This table provides consolidated descriptive values to complement the inferential statistics presented in Tables 1 and 2, ensuring transparency and reproducibility of the reported analyses.

| Domain | Group | Pre-test (mean ± SD) | Post-test (mean ± SD) | Follow-Up (mean ± SD) | Δ Post→Follow‑Up ( 1 month) | % Change |
| --- | --- | --- | --- | --- | --- | --- |
| Knowledge | CI | 2.00 ± 0.00 | 2.71 ± 0.49 | 2.29 ± 0.49 | −0.42 | −15.5% |
|  | VR | 1.86 ± 0.38 | 2.29 ± 0.49 | 2.00 ± 0.58 | −0.29 | −12.7% |
|  | CI+VR | 1.83 ± 0.41 | 2.50 ± 0.55 | 2.33 ± 0.52 | −0.17 | −6.8% |
| Understanding | CI | 4.57 ± 0.98 | 6.00 ± 0.00 | 5.71 ± 0.76 | −0.29 | −4.8% |
|  | VR | 3.71 ± 0.76 | 5.71 ± 0.76 | 5.71 ± 0.76 | 0.00 | 0.0% |
|  | CI+VR | 5.00 ± 1.10 | 6.00 ± 0.82 | 5.67 ± 0.82 | −0.33 | −5.5% |
| Application | CI | — | 6.00 ± 3.00 | 6.43 ± 2.70 | +0.43 | +7.2% |
|  | VR | — | 4.71 ± 2.36 | 5.57 ± 2.70 | +0.86 | +18.3% |
|  | CI+VR | — | 4.50 ± 2.51 | 5.00 ± 3.10 | +0.50 | +11.1% |

Note: Δ Post→Follow‑Up (1 month) and % Change values indicate retention or improvement between Post‑test and one‑month Follow‑Up. This table provides a stable comparative overview of group‑level performance across domains.

*Supplementary Table S2.*

Individual participant trajectories across Knowledge, Understanding, and Application domains. Raw scores are reported to illustrate variability and heterogeneity in learning outcomes, complementing the group-level analyses presented in Tables 1 and 2.

|  | Knowledge  Pre-test | Knowledge  Post-test | Knowledge  Follow-Up | Understanding  Pre-test | Understanding  Post-test | Understanding  Follow-Up | Apply  Pre-test | Apply  Follow-Up |
| --- | --- | --- | --- | --- | --- | --- | --- | --- |
| CI 1 | 2.00 | 3.00 | 2.00 | 4.00 | 6.00 | 6.00 | 9.00 | 9.00 |
| CI 2 | 2.00 | 3.00 | 2.00 | 4.00 | 6.00 | 6.00 | 3.00 | 9.00 |
| CI 3 | 2.00 | 3.00 | 2.00 | 6.00 | 6.00 | 6.00 | 3.00 | 9.00 |
| CI 4 | 2.00 | 2.00 | 2.00 | 4.00 | 6.00 | 6.00 | 3.00 | 3.00 |
| CI 5 | 2.00 | 3.00 | 2.00 | 4.00 | 6.00 | 6.00 | 9.00 | 3.00 |
| CI 6 | 2.00 | 2.00 | 3.00 | 4.00 | 6.00 | 4.00 | 9.00 | 6.00 |
| CI 7 | 2.00 | 3.00 | 3.00 | 6.00 | 6.00 | 6.00 | 6.00 | 6.00 |
| mean | 2.00 | 2.71 | 2.29 | 4.57 | 6.00 | 5.71 | 6.00 | 6.43 |
| SD | 0.00 | 0.49 | 0.49 | 0.98 | 0.00 | 0.76 | 3.00 | 2.70 |
|  |  |  |  |  |  |  |  |  |
| VR 1 | 2.00 | 2.00 | 3.00 | 4.00 | 6.00 | 6.00 | 9.00 | 6.00 |
| VR 2 | 2.00 | 2.00 | 2.00 | 4.00 | 4.00 | 4.00 | 6.00 | 6.00 |
| VR 3 | 1.00 | 2.00 | 1.00 | 4.00 | 6.00 | 6.00 | 6.00 | 9.00 |
| VR 4 | 2.00 | 2.00 | 2.00 | 4.00 | 6.00 | 6.00 | 3.00 | 3.00 |
| VR 5 | 2.00 | 2.00 | 2.00 | 4.00 | 6.00 | 6.00 | 3.00 | 9.00 |
| VR 6 | 2.00 | 3.00 | 2.00 | 4.00 | 6.00 | 6.00 | 3.00 | 3.00 |
| VR 7 | 2.00 | 3.00 | 2.00 | 2.00 | 6.00 | 6.00 | 3.00 | 3.00 |
| mean | 1.86 | 2.29 | 2.00 | 3.71 | 5.71 | 5.71 | 4.71 | 5.57 |
| SD | 0.38 | 0.49 | 0.58 | 0.76 | 0.76 | 0.76 | 2.36 | 2.70 |
|  |  |  |  |  |  |  |  |  |
| CI+VR 1 | 2.00 | 3.00 | 3.00 | 6.00 | 6.00 | 6.00 | 3.00 | 6.00 |
| CI+VR 2 | 2.00 | 3.00 | 3.00 | 6.00 | 6.00 | 6.00 | 6.00 | 9.00 |
| CI+VR 3 | 2.00 | 3.00 | 2.00 | 6.00 | 6.00 | 6.00 | 9.00 | 3.00 |
| CI+VR 4 | 2.00 | 2.00 | 2.00 | 4.00 | 6.00 | 6.00 | 3.00 | 6.00 |
| CI+VR 5 | 1.00 | 2.00 | 2.00 | 4.00 | 6.00 | 4.00 | 3.00 | 0.00 |
| CI+VR 6 | 2.00 | 2.00 | 2.00 | 4.00 | 6.00 | 6.00 | 3.00 | 6.00 |
| mean | 1.83 | 2.50 | 2.33 | 5.00 | 6.00 | 5.67 | 4.50 | 5.00 |
| SD | 0.41 | 0.55 | 0.52 | 1.10 | 0.00 | 0.82 | 2.51 | 3.10 |

Raw scores illustrate variability within and between groups. Application scores showed wide ranges (CI 3–9, CI+VR 0–9, VR 3–9). Learners with lower Post‑test scores often demonstrated large relative improvements at Follow‑Up, while higher scorers plateaued or declined. Group means are reported in the main text (Tables 1–3).

*Additional file 1:* *Pre and Post Test Examination:*

Fundamental Knowledge of Normal Anatomy Related to Dental Implant Planning

Level: Third-Year Dental Students

Multiple Choice (100%) + Short Answer (100 %)

Scoring

All scores in each domain are transformed into rubric scores from 0–3:

- 0 = 0 %
- 1 = 1–50 %
- 2 = 51–99 %
- 3 = 100 %

Impact factors:

- Knowledge = 1
- Understanding = 2
- Application = 3

*Part I*: *Multiple Choice Questions (Items 1–7)*

*(for assessing knowledge)*

1. Which of the following anatomical structures is located near the anterior region of the maxilla and requires special caution during implant placement?

A. Inferior alveolar nerve

B. Mental foramen

C. Incisive canal

D. Mandibular canal

2. Which anatomical structure is most important to consider when planning implant placement in the posterior mandible?

A. Maxillary sinus

B. Mandibular canal

C. Zygomatic arch

D. Nasal floor

3. In the maxilla, implant placement in the posterior region requires careful assessment of its relationship with which structure?

A. Mental foramen

B. Maxillary sinus

C. Submandibular fossa

D. Genial tubercle

4. What is the recommended minimum bone height for routine dental implant placement?

A. 2 mm

B. 4 mm

C. 6 mm

D. 10 mm

5. Which of the following landmarks is commonly used to identify the position of the mental foramen on panoramic radiographs?

A. Ramus of mandible

B. Mandibular notch

C. Apex of the second premolar

D. Lateral incisor

6. What is the typical characteristic of the alveolar bone in the anterior mandibular region?

A. Thick and dense

B. Thin and inclined lingually

C. Thick and buccally inclined

D. Thin and buccally inclined

7. Which of the following is not an anatomical factor influencing the success of dental implant placement?

A. Bone height

B. Mucosal thickness

C. Patient preference

D. Bone density

*Part II:* *Short Answer Questions (Items 8–10)*

*(for assessing understanding)*

8. Explain the importance of evaluating the position of the mandibular canal prior to implant placement in the posterior mandible.

9. Identify two important anatomical structures relevant to implant placement in the anterior maxilla and explain why they must be considered.

10. Describe basic management strategies when there is insufficient bone volume for implant placement in the posterior maxilla.

*Additional file 2: Clinical application test*

*Question 1*

This patient requires a dental implant at tooth #37. The cross-sectional view of the intended implant site, including measured bone height and width, is shown in the image.


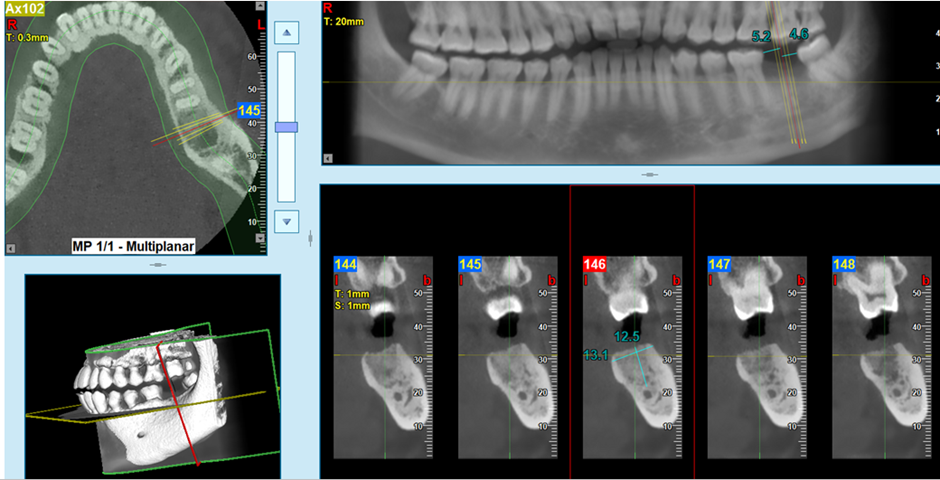


From the cross-sectional image below, if you think implant placement is feasible, select the appropriate implant size and draw the implant within the bone.


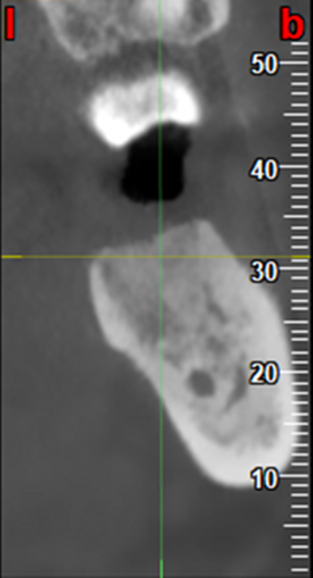


Please consider safety margins and ensure optimal implant function.

Available implant sizes:

H = 8 mm, W = 4 mm

H = 12 mm, W = 5.5 mm

H = 10 mm, W = 4 mm

If you think the implant cannot be placed, please explain your reasons:

…………………………………………………………………………………………………………………………………………………………………………………………………………………………………………………………………

*Question 2*

This patient requires a dental implant at tooth #23. The cross-sectional view of the intended implant site, including measured bone height and width, is shown in the image.


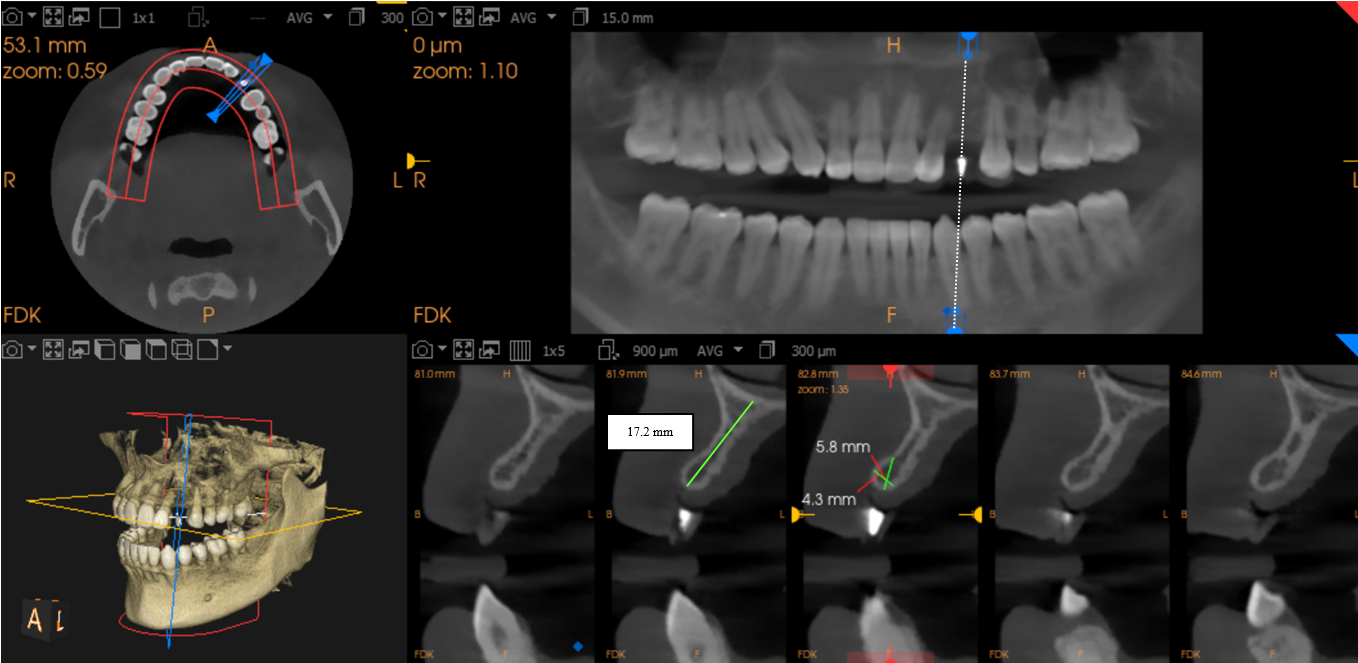


From the cross-sectional image below, if you think implant placement is feasible, select the appropriate implant size and draw the implant within the bone.


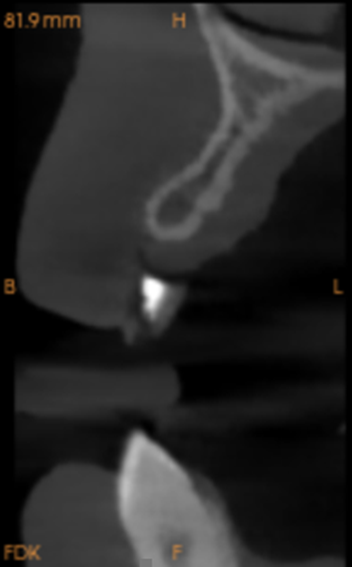


Please consider safety margins and ensure optimal implant function.

Available implant sizes:

H = 10 mm, W = 3.3 mm

H = 12 mm, W = 4.1 mm

H = 8 mm, W = 3.3 mm

If you think the implant cannot be placed, please explain your reasons:

…………………………………………………………………………………………………………………………………………………………………………………………………………………………………………………………………

*Additional file 3: Interview Guide*

Students were asked to reflect on their experiences with the instructional modality using the following prompts:

1. How did you perceive the learning activity overall?
2. In what ways did it differ from your previous coursework?
3. Which aspects of the activity did you find most valuable?
4. Were there elements that felt unclear or could be improved?
5. Did the activity enhance your understanding of anatomy?

For the CI+VR group, two additional prompts were included to capture perceptions of the combined modality:

1. What aspects of learning with both CI and VR did you find most valuable
2. What challenges or drawbacks did you experience when learning with CI and VR?
